# Considering risk contexts in explaining the paradoxical HIV increase among female sex workers in Mumbai and Thane, India

**DOI:** 10.1186/s12889-016-2737-2

**Published:** 2016-01-28

**Authors:** Sunita V S Bandewar, Shalini Bharat, Anine Kongelf, Hemlata Pisal, Martine Collumbien

**Affiliations:** 1 Independent Senior Research Professional in Bioethics, Global Health, and Program Evaluation, Pune, MH 411 008 India; 2 School of Health Systems Studies, Tata Institute of Social Sciences, Mumbai, MH India; 3 Present address: Norwegian Red Cross, Vestlia 4, Jessheim, Norway; 4 Independent Senior Research Professional, Pune, MH India; 5 London School of Hygiene and Tropical Medicine, 15-17 Tavistock Place, London, UK

**Keywords:** Risk context, Female sex work, Targeted interventions, Avahan, India, HIV prevention, Unintended intervention effects

## Abstract

**Background:**

The period 2006–2009 saw intensive scale-up of HIV prevention efforts and an increase in reported safer sex among brothel and street-based sex workers in Mumbai and Thane (Maharashtra, India). Yet during the same period, the prevalence of HIV increased in these groups. A better understanding of sex workers’ risk environment is needed to explain this paradox.

**Methods:**

In this qualitative study we conducted 36 individual interviews, 9 joint interviews, and 10 focus group discussions with people associated with HIV interventions between March and May 2012.

**Results:**

Dramatic changes in Mumbai’s urban landscape dominated participants’ accounts, with dwindling sex worker numbers in traditional brothel areas attributed to urban restructuring. Gentrification and anti-trafficking efforts explained an escalation in police raids. This contributed to dispersal of sex work with the sex-trade management adapting by becoming more hidden and mobile, leading to increased vulnerability. Affordable mobile phone technology enabled independent sex workers to trade in more hidden ways and there was an increased dependence on lovers for support. The risk context has become ever more challenging, with animosity against sex work amplified since the scale up of targeted interventions. Focus on condom use with sex workers inadvertently contributed to the diversification of the sex trade as clients seek out women who are less visible. Sex workers and other marginalised women who sell sex all strictly prioritise anonymity. Power structures in the sex trade continue to pose insurmountable barriers to reaching young and new sex workers. Economic vulnerability shaped women’s decisions to compromise on condom use. Surveys monitoring HIV prevalence among ‘visible’ street and brothel-bases sex workers are increasingly un-representative of all women selling sex and self-reported condom use is no longer a valid measure of risk reduction.

**Conclusions:**

Targeted harm reduction programmes with sex workers fail when implemented in complex urban environments that favour abolition. Increased stigmatisation and dispersal of risk can no longer be considered as unexpected. Reaching the increasing proportion of sex workers who intentionally avoid HIV prevention programmes has become the main challenge. Future evaluations need to incorporate building ‘dark logic’ models to predict potential harms.

## Background

The response to India’s HIV epidemic with the large scale-up of prevention efforts has been well documented in the last decade [1]. Both the National AIDS Control Program (NACP) and the Bill and Melinda Gates Foundation -through its flagship programme Avahan- have implemented targeted interventions among groups at higher-risk, including female sex workers and their clients, men having sex with men and injecting drug users [2].

Avahan’s standardised service package, implemented through NGOs, has included the delivery of peer-based education focusing on condom use and enhanced access and uptake of sexually transmitted infection (STI) screening and treatment [1]. Linking changes in the prevalence of HIV and other STIs, sexual risk and risk reduction practices to intervention exposure has been central to Avahan’s impact evaluation. These changes were measured between 2 rounds of Integrated Behavioral and Biological Assessments (IBBA) surveys in 2006 and 2009 among key population [3]. Among female sex workers in the state of Maharashtra, HIV prevalence increased slightly between 2006 and 2009 (from 26 % to 27.5 %), despite an increase in intervention coverage from 28 % to 66 % and a reported rise in condom use from 76 % to 95 % [3]. The increase in HIV prevalence was statistically significant in the two neighbouring districts of the metropolis, Mumbai and Thane, where it rose sharply from 24 % in 2006 to 31 % in 2009. Among women who reported entering sex work within the last year, HIV increased from 8 % to 16 % in Thane while remaining at 19 % in both rounds in Mumbai [3]. The IBBA is the only independent source of HIV estimates among brothel and street-based FSWs and with the two survey rounds consistently implemented, we found no technical or methodological reason to doubt the veracity of the HIV estimates. In both rounds about 400 sex workers were sampled in each strata (street-based and brothel-based) and each district [3]. While survey implementation was comparable across rounds, discussions with IBBA field teams revealed substantial changes in the sampling ‘universe’ with a reduction in the number of brothels, and the number of sex workers in each brothel. The size of both brothel and street-based sex worker populations diminished, especially in Mumbai [4] suggesting a substantial shift in the composition of the sex worker population.

The change in the sampling context provided a compelling reason for studying wider changes in the sex work environment as public health interventions have been shown to lead to unintended effects and cause potential harm when complex social systems are interrupted [5]. Extensive urban redevelopment has characterised Mumbai’s rapid transition from an industrial hub to a financial centre dominated by the service sector [6]. The constraining effect of context on individual agency to reduce harm have long been recognized in HIV prevention [7]. Yet empirical understanding of how scale-up of HIV prevention interacts with sex workers’ risk environment in Mumbai to explain the rise in HIV prevalence has so far been missing. This paper reports on a qualitative study designed to help interpret these unexpected observations considering a risk environment framework [8] to go beyond individual risk. Our analysis makes an important contribution to documenting adverse impacts of public health interventions, so that they might be avoided in future.

## Methods

The extensive literature on the changing political economy of Mumbai and land redevelopment policies informed the design of our qualitative study to explore how the broader context shapes the organization of the ‘sex work industry’, the vulnerability of FSWs, and HIV risk. In total, we conducted 36 individual interviews, nine joint interviews, and ten focus group discussions (FGDs). Through these, we interacted with 140 individuals (82 women and 58 men) associated with HIV prevention interventions. Participants came from diverse sites within Mumbai and Thane, with a few research participants from one other city in Maharashtra in order to understand the challenges of FSW mobility. We used purposive sampling, starting from an initial list of organisations and individuals representing key constituencies relating to HIV prevention work. We sought study participation from both government and non-government players and those working with diverse typologies of sex workers. We interacted with 67 women in sex-work (of which 63 working on prevention) and nine clients of sex-workers. Key informants involved in the implementation of programmes included 83 peer educators (PEs) and outreach workers (ORWs) at the grassroots level, seven program coordinators (PC) at mid-level and 21 senior staff in leadership positions, of which three were sexual rights advocates. We also interviewed one politician, one person from the police department, a police informer, a taxi driver, an intervention researcher and seven service providers.

We stopped interviewing when interviews ceased to offer additional insights into the topic of enquiry. Most fieldwork was done between mid-March and mid-May 2012, with some supplementary interviews with participants to follow up emerging themes.

We used culturally sensitive methods of seeking informed consent from research participants. We adopted a two-staged approach to contact sex workers and clients since access was through organisations or individuals working with them. Time and location of interview was the choice of the participants and interviews were strictly private. Written consent was obtained before the start of each interview. Interviews were conducted in Hindi, Marathi or English depending upon participants’ preferences. FGDs were conducted in Hindi or Marathi. Interviews were digitally recorded and notes taken in the few instances in which consent for recording was not given. Recordings were transcribed and the Marathi and Hindi ones translated into English, and coded for in-depth analysis using Atlas Ti (Version 7.0). Translation and interpretation bias was minimized by collective engagement of the authors with the data. The two authors who conducted the interviews (SVSB and HP) remained closely involved in transcription and translation processes and the second author (SB) validated transcripts.

Data collection was iterative with team discussions and analysis shaping the content of subsequent interviews. Our assumptions about structural changes affecting the risk environment served in developing the initial coding framework. About 82 code families were created using Atlas ti. The emerging themes and analytical categories with sample representative quotes were delineated in an excel spread sheet. This material was used for in-team consultative meetings and helped to internally validate data interpretations, and to identify gaps and contradictions, if any. We refined analysis through three subsequent iterations to develop this manuscript as a “best fit” for the data.

The research proposal was reviewed by the Institutional Ethics Committee, Tata Institute of Social Sciences (TISS), Mumbai, India and the IRB from the London School of Hygiene and Tropical Medicine. Standard practices in compliance with the key research ethics guidelines were followed: seeking informed consent of prospective research participants and ensuring privacy and confidentiality. In FGDs, confidentiality could only be maintained to the extent that all participants co-operated with the request to keep the proceedings confidential. Care was taken to protect identities of individuals by requesting participants to not mention names of individuals. Digital interviews, transcripts, and any other information relating to research participants was pass-word protected and access restricted to research team members and associates only. Since the confidentiality clause was included in our consent procedure, data cannot be made publicly available for ethical reasons. Necessary precaution was taken to protect research participants’ identities when using quotes, identifying them only by their role in the implementation of HIV prevention and an interview number.

## Results

### Urban redevelopment

Dramatic changes in Mumbai’s urban landscape dominated participants’ accounts, with dwindling sex worker numbers in traditional brothel areas attributed to urban restructuring. Land prices in Kamathipura -the main red-light district in South Mumbai - had soared, attracting private builders and land developers.

“If the towers come up here, it will fetch crores of rupees [1 crore = $160,000] to owners and builders.” [PC (20)].

Participants reported that landlords, keen on deals with the builders, offered large lump sums to brothel owners to vacate premises and leave. Often in perpetual debt, these brothel owners were said to find these sums difficult to resist.

However the brothel-based sex workers themselves did not receive such windfalls and migrated to suburban Mumbai, affecting the size of the visible sex workers population in the main red-light areas.

“Three years ago around 20,000 sex workers used to do business in the daytime from these ten main sex work hubs from South Mumbai. Now you will not even find 2000 sex workers there. Builders are trying hard to evict them. [Head and PC, CBO (16)].

### Gentrification

A taxi driver (55) aptly described how gentrification contributed to further dispersal of sex work:

“Because of good families who came here to stay in the new towers which got constructed by demolishing old buildings, all dirt from the area got cleaned (*gandagi saaf ho gai yahanse*). Because of their complaints police closed down that dirty work in their area and now sex work does not take place on their roadside.”

Participants saw street-based and brothel-based sex work cleared from what were becoming prime neighbourhoods, and police acting on behalf of the new residents.

“Now because of the police girls are not able to stand on the road. If police see the girl, they charge her 1250 rupees [$20]” [ORWs & PEs (23)].

### Anti-trafficking

An escalation in police raids was reported by many participants, and seen to be brought about by both gentrification and anti-trafficking efforts.

“They [police] are putting raids for the reasons of girls from Bangladesh, for minor girls, girls from other states who are brought here in this business [of sex-work]. So we cannot say that raids are happening because of builders’ lobby alone.” [PC (19)].

Underage sex workers are known to be kept out of view by brothel owners

“They do not bring below 18 girls in front. Every brothel has some place to hide the girls …” [Police informer (10)].

Raids can bring the business to a complete halt affecting all sex workers as police lock the entire building.

### Change in management of sex trade

In order to avoid raids, pimps and madams were reported to increasingly control women in smaller pockets, away from main red light areas. As customers were always in search of new faces, sex workers were circulated across Mumbai and Thane, a practice facilitated by modern communication technology. Another business tactic which increased mobility was the rise in relatively well-paid contract work.

“The rate is fixed based on the girl’s looks, how many clients she can take and what is her age. Contractor should also be able to make the profit. If girl takes four clients in daytime and one in the night then she gets 30,000 rupees. Half of the money she gets before going on contract and half after she completes the tenure.” [ORWs & PEs (18)]

The emergence of affordable and accessible mobile phone technology did not only facilitate brokers and pimps to adapt to changing contexts it also enabled the more independent sex workers to trade in more hidden ways.

### Diversification

Apart from the dispersal of the traditional brothel and street-based sex workers, the population of women providing sexual services was seen as becoming increasingly diversified. Bar girls lost their relatively safe and lucrative livelihoods following a ‘bar dance ban’ invoked throughout Maharashtra in August 2005. Many now needed to generate income by selling sex.

“… Because of the ban we [bar girls] have to sell our rooms, our jewellery; we are facing lot of problems. Circumstances have made us to do what we did not wish to do [sex-work]. Previously we used to take one week just to tell our name to the customer. Now when a bar girl walks on the road, looking at man, at the back of her mind, she thinks - this is a rich person, he should come to me. … We used to think several times before taking one customer in a month. Now we take four customers in a day. Earlier what we used to earn through dance now we are not getting by sleeping.” [ORWs & PEs, (23)].

Increased economic hardship was the common motivation for housewives and daily labourers needing to secure additional income by selling sex, either because of their partners’ unemployment and/or families’ financial need.

“Addictions, uncertainty of industries, factory closures, and recession causes unorganized sex work. It is also because of globalization, pushing into the unorganized sector is clearly a fall out.” [Head & Sr staff, NGO (37)]

Numerous examples were given of women labourers in construction or the loom industry exchanging sex in order to secure further paid work. These women sell sex on a part or full-time basis and remained hidden to the interventions.

“During economic slag such type of sex work has increased tremendously. We do not have their registration so we cannot highlight it.” [PCs, (11)]

### Stigmatisation

Some attributed the diversification of sex work, at least partly, to the HIV interventions’ emphasis on reducing the risk of HIV transmission from sex workers, which led to stigmatisation

“People have targeted [brothel-based] FSWs. They think that, they may get HIV infection by going to FSW” [PEs & PCs (49)].

For HIV positive sex workers knowledge of their status was stated as a reason why women move to different areas.

“Sometimes when sex worker’s positive status gets disclosed, she leaves her areas and moves to another hub.” [PC, (20)].

Having considered various structural influences on the sex workers’ immediate work and social environment we now turn to how this may have influenced women’s agency to adopt or maintain safe behaviours.

### Condom use as a social norm

A relentless intervention focus on condom use may have increased social desirability in reporting safer sex. Participants stressed that the ‘condom message’ was continually reiterated

“We hammer the information on those who are registered with us and always ask them if money was more important than their life” [PCs, (35)].

Condom use in commercial sex thus increased substantially and there has been a resounding change in norms around use with many claiming that sex workers do not have sex without condoms.

### Deliberate non-use of condoms

Yet numerous examples were given of women compromising on its use across all strata of sex workers. The rationale for non-use was essentially financial as many sex workers had acute levels of economic hardship and debt. Despite full awareness of health risks, the distant threat of early death through disease is easily discounted by a more immediate need for money.

This leads us to question the veracity of condom use reported by sex workers, as normative reporting may well be the flipside of strong normative behaviour change.

“Sometimes what happens even though sex worker is not using condom, she will say that she is using condom. We give them information but how we will come to know what she does inside the room?” [PCs and ORWs (19)]).

### Condom fatigue

The reiteration of the ‘condom message’ seems to have led to information fatigue. Deliberate misreporting was illustrated when several participants talked about the implementation challenges of ‘using double condom’. A senior implementer with long experience in HIV prevention among brothel-based FSWs was doubtful:

“Because of the intervention (…) now women are aware about what these AIDS people will ask and they started giving stereotype answer after two years of basic intervention so what started happening was, whenever any staff peer educator, counsellor or ORW goes to the brothel and asks, do you use condom with each customer? They say yes we use condom with every customer, they can give demonstration of condom. On top of that they started telling us that they use 2 condoms at a time as if one condom breaks then we will be at risk so we use two condoms at a time. After few years they have started saying that they use 3 condoms at a time. So what was it? We have not taken any hint at that time also, that they need something more now.” [Senior staff, NGO (32)].

Further, she diagnosed fear of stereotyping as the underlying cause of this subversion and criticised the programme’s simplistic response (ie. insisting on the use of single condom) as symptomatic of its inability to understand, respond and adapt to the needs of FSWs on the ground. Again misunderstood as flawed strategies of protection, this subversion was ironically countered with even more information on correct use.

### Riskier practices

Widespread access to pornography influenced clients’ aspirations to experiment with ‘unconventional’ sex.

“People download the English blue films in the mobile. They see it and want to have such type of sexual pleasure” [Former client (40)].

An increase in demand for oral and anal sex with higher financial returns for the woman was reported by many. With these practices far from normalised, condoms were unlikely to be used. While hardship made women deliberately forego condom use, there also seemed a lack of awareness of HIV risk associated with anal sex.

### Gatekeepers preventing condom use

There was overwhelming evidence that sex workers’ agency to choose condoms was still compromised by gatekeepers, especially for women new to the sex-trade. Young girls are still sold to brothel owners, who continue to “own” and control them. In a challenging economic climate, encouraging girls to provide sexual services without condom becomes a survival strategy for brothel owner as she herself pays more to police and to local *goondas*. As long as the *gharwali* has not recovered her investment she deliberately restricts their girls’ exposure to the HIV prevention interventions.

“Actually when the [sex worker] is new in the business she needs information about condom. But that time brothel owner does not allow her to come in front of us. By the time she repays all her loan she has become 2–3 years old [in sex work] and sometime positive also.” [PC, ORWs, and PEs, (19)].

The delay in the interventions reaching the most vulnerable young girls with information on HIV risks and condom negotiation skills is obvious and simple: outreach workers are bad for business. By design current targeted interventions do not reach out to these younger women and there is one niche in the sex work market in which condoms are never used: when girls are presented as virgins to wealthy clients under a practice called ‘sar dhakai’.

“Yes, at the time of breaking seal (virginity) the condom is not used. For that they pay INR 40,000 to 50,000 [about USD 1000/-] for [being with the girl for] 24 hours. They keep the girl for one or two days with them, and do the sex with her by drinking liquor. They [clients] think that since the girl is ‘seal pack’ she does not have HIV infection. So they do not use condom.” [PE, (6)].

The high premiums paid to the brothel owners, and sufficient demand perpetuates the practice. This deliberate withholding of condoms, safer sex knowledge and negotiation skills may explain the high HIV prevalence observed among ‘new’ sex workers. Yet, there was also evidence that FSWs misreport themselves as ‘new’ to sex-work.

**“**They [FSWs] tell us ‘I am new; I started sex work one month back, two months back.’ They lie to us. When they test [HIV] positive we realise that they were already positive before coming here.” [ORWs and PEs, (45)].

### Increasing dependence on lovers

Lastly, participants suspected HIV transmission through non-commercial encounters. More FSWs were believed to develop intimate relationships with men whom they refer to as ‘lovers’, ‘pyarwala’ or ‘husbands’. Their emotional desire for an intimate relationship can be appreciated given women’s pressing vulnerable state, looking for protection and support, and responding to strong societal norms around marriage, or anything that resembles it. With the emotional investment and looking for commitment, condoms undermine these relationships.

“It is problematic to use condom with one’s regular partner as then her commitment is questioned.” [PC, (25)].

These relationships carry HIV risk as a man can be the ‘lover’ of several women. However, the emotional investment may not only be from women’s side

“I have an HIV positive sex worker … even when she knows her status and after we have counselled her, she does not use condom with her regular partner. He knows about the fact that she is positive; … he told us that because he loved her he wouldn’t use condom [with her].” [Head, CBO (15)]

Despite the explicit risk of HIV transmission, love was seen as incompatible with condom use.

## Discussion

We illustrated various social and structural changes in Mumbai’s urban environment which impacted the immediate risk context and vulnerability of sex workers. In contextualising our findings with the wider literature on urban redevelopment, we explain how the social conditions of the sex trade mediate the larger economic, political and legal structures, leading to the three unexpected observations of declining numbers of ‘visible’ sex workers, their increasing HIV prevalence, and high and increasing levels of HIV among ‘new’ sex workers. Throughout we consider the effects on the generalisibility and validity of the IBBA estimates of risk reduction and HIV prevalence.

Firstly, exacerbated marginalisation, forced eviction and migration have unmistakably led to a reduction in ‘visible’ brothel- and street-based sex workers. Compelling narratives of forced dispersion of the sex trade were attributed to rapid urban redevelopment and increased gentrification of the traditional red light brothel areas. The literature on Mumbai redevelopment policies confirms that real estate values in the red-light districts soared given the central location between the business area in South Mumbai and the residential suburbs in the North [9]. This started in the 1990s as India’s economic policies shifted from a highly regulated economy towards a free-market one [10]. Neoliberal ideology affected governments’ housing and urban restructuring policies in fundamental ways, allowing the involvement of private developers, builders, major banks and international capital [11]. Developing a global financial centre kept the focus on improving infrastructure to make Mumbai attractive to international investors, with little attention to the social issues of the urban poor [12]. Indeed, our data exposed how economic hardships in slums compelled women into sex work. The increased use of mobile phones to solicit sex, another force of globalisation, was used by women who could gain more independence and anonymity.

The persistent focus of the intervention on condom use with sex workers has inadvertently contributed to the dispersal and diversification of the sex trade as clients avoid the more visible sex workers, seeking out women who operate in a more subtle and hidden ways (as discussed in more depth elsewhere [13]). Heightened animosity against sex workers by new residents emerged too, with populist support for their eviction implicit in the media coverage on 50 % of brothels in south Mumbai having been shut down: “Though there had been a rise in murder, extortion and other serious crimes in 2010, the police seem to have managed to curb the menace of flesh trade” [14]. Intense gentrification changed the social fabric and political climate in the city in what some have described as class cleansing or “revanchism” [10, 15, 16]. Many NGOs representing a fragmented majority of the marginalised poor lose out against other citizen groups who represent the minority of middle-class people who campaign against encroachment of public spaces [17].

The escalation in police raids was attributed to both gentrification and abolition efforts. Relying on state control to restore morality, there was increasing high-handed implementation of the Immoral Traffic Prevention Act (ITPA), an act in existence since 1956 [18]. Brothel owners started to suspect outreach workers from collaborating with the police, affecting the credibility of genuine harm reduction efforts [13]. Forced dispersion has thus come with a heavy cost to public health as women de-prioritise health concerns for anonymity and economic necessities, similar to what followed forced demolition of red light area in Goa [19]. The same ITPA act was used in 2005 to invoke the bar dance ban in Maharashtra leading dancing girls to lose their livelihoods with some starting to covertly sell sex, as evidenced in our data.

Seemingly distant policies and laws have thus profoundly shaped the risk environment of all women selling sex, not just the visible ones. Driving the sex trade underground does have direct implications for monitoring risk and HIV as the IBBA sampled visible sex workers only. Whereas both the 2006 and 2009 IBBA surveys have captured representative samples of brothel and street-based sex workers, these visible groups have increasingly become less representative of all women selling sex.

Second, we turn to the paradoxical increase in HIV prevalence among the visible sex worker population. Our data suggests that the effect of global and structural forces is mediated through economic vulnerability shaping women’s decisions to engage in more risky practices for higher returns. Client demand for unprotected sex is sustained as only 40 % of clients of brothel-based sex workers in Maharashtra reported consistent condom use in the 2009 IBBA [20], seriously challenging the veracity of 95 % condom use reported by FSW [3]. This level of normative reporting is feasibly explained by the interventions’ relentless emphasis on condom use, and women’s resulting fatigue, annoyance and rebellion against it as demonstrated by their claimed use of two or three condoms. The ultimate evidence of non-use seems to be the undeniable increase in HIV prevalence. Hence self-reported condom use is no longer a valid indicator of risk reduction.

The recent rise in anal intercourse, associated with clients’ ready access to pornography on smart phones can be seen as another force of globalisation. The practice is largely unprotected and has been overlooked in HIV prevention programming with FSWs [21]. Intensified stress and insecurity stemming from professional isolation and the disruption of their social context increased the women’s dependency on intimate relationships for emotional and instrumental support, with concepts of increased risks next to meaningless. Sex workers reporting intimate partnerships increased from 30 % in 2006 to 50 % in 2009, with condom use as low as 11 % in 2009 [4].

Study participants stressed mobility-related vulnerability linked to the general dispersal of the sex trade, an increase in contract work and stigmatisation. Stigma reportedly drove HIV positive sex workers to new locations in order to conceal their status. This needs to be considered as one of the explanations for HIV increasing as measured in the IBBA. We believe that the hostile risk context we described will lead this HIV-related mobility to be quite circular in nature, and hence not the main explanation. Nevertheless, anonymity afforded by the fluid and fast moving nature of the sex trade in Mumbai may have contributed to the higher prevalence observed in 2009. The wider literature confirms HIV-related mobility driven by stigma [22] and particularly high mobility among FSW in Thane (42 %) and Mumbai (33 %) [23]. In terms of contract work, the fear and threat of violence in new locations affects FSW’s power in negotiating condom use [24] with forced non-condom use in Andhra Pradesh reported by 41 % FSW, this was not dissimilar for women not under contract [25].

Lastly, there are both the young and ‘new’ sex workers showing high and rising levels of infection. Despite dissipating brothels, the power structures of the sex trade remained intact, staunchly ‘protecting’ new entrants from interacting with outreach workers. Mumbai is a prime destination for trafficked women coerced into sex work [26] and the stronghold of *sar dhakai* seems undeniable. When a relatively closed network of wealthy customers pay for sex with ‘virgins’, modelling predicts high transmission once HIV is introduced [27]. In the whole of Maharashtra, HIV prevalence increased significantly from 9 % to 23 % among 18–20 years old FSW in the 3 years between IBBA rounds [3]. Harm reduction programmes do fail the subgroup of coercively held young women [28]. Yet our study confirms the harm caused by police raids, instigated by the rehabilitation ideology of sexual exploitation. It not only fails to reduce both exploitation and sex work, it undermines prevention. Anti-trafficking efforts can be and have been aligned with HIV prevention where strong peer networks are committed to self-regulation [29, 30]. However, sex worker communities in Mumbai have further destabilised as the intervention interacted with the disrupted dynamic context in which many FSW decided to seek protection from prevention programmes and actively avoid attempts to mobilise the community of sex workers [13].

Not all recent entrants in sex work are young or coerced, nor are they all introduced via the brothel system, and our data showed that not all are even ‘new’. In Karnataka, it took women on average 8.8 years to self-identify as sex workers [31]. In Mumbai, resistance to the stigmatising label of ‘sex worker’ [13] seems to result in underreporting of duration in sex work and thus spuriously inflate the HIV prevalence among new entrants as reported in the IBBA.

Potential harm has been given insufficient consideration in Avahan’s impact evaluations, with a too narrow focus on indicators of risk reduction, ignoring even radical changes in sampling frame. The strength of this study is the focus on the dynamic risk context to provide plausible explanations for paradoxical observations including the effect on the validity and generalisability of the IBBA data to evaluate impact. A study limitation is that the fieldwork was done in 2012, nearly 3 years after the second IBBA measured the increased HIV prevalence. Yet most of the documented changes are ongoing historical processes linked to globalisation, many of them starting well before 2006 and still ongoing.

## Conclusion

Targeted individuals respond to HIV prevention programmes which interact with various contextual factors to impact the dynamic risk context in which HIV gets transmitted. HIV prevention is inherently political [7] and when we implement ostensibly value-neutral harm reduction programmes in contexts that strongly support abolition of sex work, harm does ensue. In Mumbai, power structures in the sex trade remained insurmountable barriers and criminalization and law enforcement undermined and annihilated the persuasion and empowerment principles of harm reduction. It is time to re-evaluate the way HIV prevalence is estimated in rapidly changing contexts. The HIV prevalence among the various hidden subgroups of sex workers is indeed unknown, but the risk context has become ever more challenging, with animosity against sex work amplified since the scale up of targeted interventions. Reaching the increasing proportion of sex workers who intentionally avoid HIV prevention programmes has become the main challenge. We can now no longer claim unintended effects as unexpected and future evaluations need to incorporate building ‘dark logic’ models [5] to predict potential harms.
